# Unusual Presentation of Embryonal Carcinoma of the Testis: A Case Report

**DOI:** 10.7759/cureus.35175

**Published:** 2023-02-19

**Authors:** Youness Tahri, Othman Moueqqit, Mohamed Mokhtari, Mohammed Ramdani, Miry Nadir, Amal Bennani, Ali Barki

**Affiliations:** 1 Urology, Mohammed VI University Hospital, Oujda, MAR; 2 Urology, Faculty of Medicine and Pharmacy of Oujda, Mohammed First University of Oujda, Oujda, MAR; 3 General Medicine, Faculty of Medicine and Pharmacy of Oujda, Mohammed First University of Oujda, Oujda, MAR; 4 Urology, Service Urologie Chu Mohamed Vi Oujda, Oujda, MAR; 5 Pathology, Mohammed VI University Hospital, Oujda, MAR; 6 Pathology, Faculty of Medicine and Pharmacy of Oujda, Mohammed First University of Oujda, Oujda, MAR; 7 Anatomopathology, Faculty of Medicine and Pharmacy of Oujda, Mohammed First University of Oujda, Oujda, MAR

**Keywords:** metastasis, lymph node, ureterohydronephrosis, testicular tumour, embryonal cell carcinoma

## Abstract

Embryonal carcinoma is a rare and aggressive type of non-seminomatous germ cell tumor that typically affects young to middle-aged individuals. It is often discovered by the patient or during routine medical exams as a painless or occasionally painful lump. Other revealing symptoms, such as lumbar pain or renal colic, are very uncommon in the literature. In this case report, we aim to highlight a case of embryonal carcinoma in a 21-year-old patient, which was discovered following the diagnostic workup of a left lumbar pain episode.

## Introduction

Embryonal carcinoma is a rare type of non-seminomatous germ cell tumor, representing only 1%-5% of all cases [1-3]. It is the second most commonly (80%) observed component of mixed germ cell tumors after seminoma [4]. This type of tumor tends to affect young to middle-aged individuals with an average age of 31-32 years [4,5]. Approximately 66% of patients with embryonal carcinoma are found to have metastasis at the time of diagnosis [5]. These tumors tend to have an aggressive course due to their early tendency to invade other parts of the body [3,1]. Early diagnosis and treatment are, therefore, critical for a positive outcome [1].

Like many other testicular tumors, embryonal carcinoma is often discovered by the patient or during routine exams as a painless or occasionally painful lump [1]. The first imaging method used for such cases is ultrasound, which typically shows a well-defined and heterogeneous lesion [1]. However, according to our research, it is rarely revealed by left lumbar pain or renal colic. We present a case of a 21-year-old patient that presented to the emergency department with acute left lumbar pain following uretero-hydronephrosis, a condition that is characterized by the dilation of the ureters and kidneys due to obstruction or reflux [6].

## Case presentation

A 21-year-old patient with a history of a leg fracture five months ago following a road accident and subsequent osteosynthesis presented to the emergency department with left lumbar pain for one month. The patient reported having already received symptomatic treatment (oral analgesia) for the same complaint three weeks previously.

Upon examination, the patient was in good general condition with normal vital signs, was afebrile and had a body mass index of 24.3 kg/m2. Clinical examination revealed left lumbar tenderness. Examination of the external genitalia revealed a solid and a painless mass in the left testicle (the patient reported some episodes of testicular heaviness), and the rest of the clinical examination was unremarkable.

The results of the complete blood count and serum biochemistry were normal (hemoglobin 12.3 g/dL; creatinine 9 mg/L), and the urinary cytobacteriological culture was sterile.

**Figure 1:**
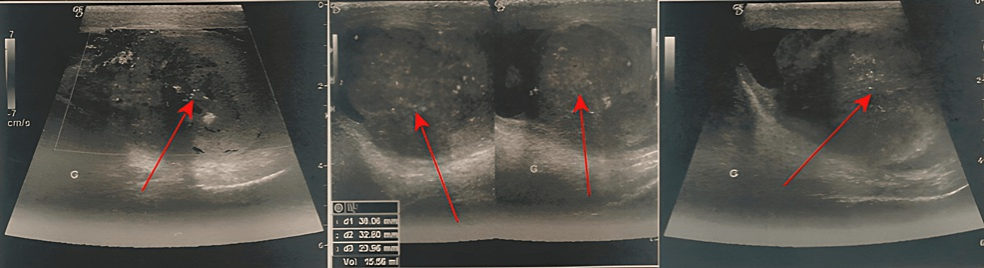
Ultrasound images showing a left testicular mass

A scrotal and renal-vesical-prostatic ultrasound (US) was performed, showing the presence of a hypoechoic 38x33x24 mm and 16 mL lesion with scalloped contours, vascularized with micro-calcifications in the left testicle, with the presence of a small liquid layer in the tunica albuginea; the right testicle was normal. There was no sign of testicular torsion. The left kidney was in its usual position, of normal size, with good parenchymal-sinus differentiation, and was the site of uretero-hydronephrosis without visible obstruction (Figure 1).

**Figure 2:**
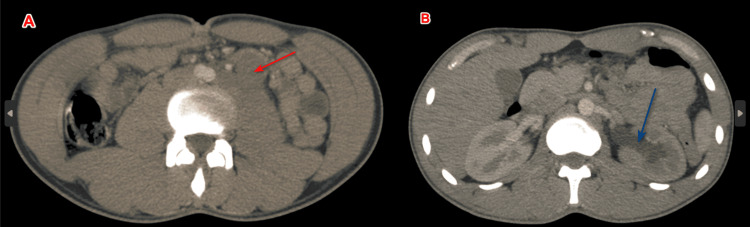
Abdominal and pelvic CT axial section showing left lateral-aortic adenopathy (red arrow) compressing the homolateral ureter (A) and responsible for uretero-pyelo-caliceal dilatation (blue arrow) (B)

In addition, a computed tomography (CT) scan was performed and revealed a large left para-aortic lymph node with a 27 mm diameter hypodense area of necrosis, compressing the adjacent ureter, resulting in upstream ureter-pelvicalyceal dilatation with delayed secretion, with no other suspicious lesions (Figure 2).

Tumor marker levels were measured: alpha-fetoprotein (AFP) and human chorionic gonadotropin (HCG) levels were normal, and lactate dehydrogenase (LDH) was slightly elevated at 267 U/L (135-225 U/L)

**Figure 3:**
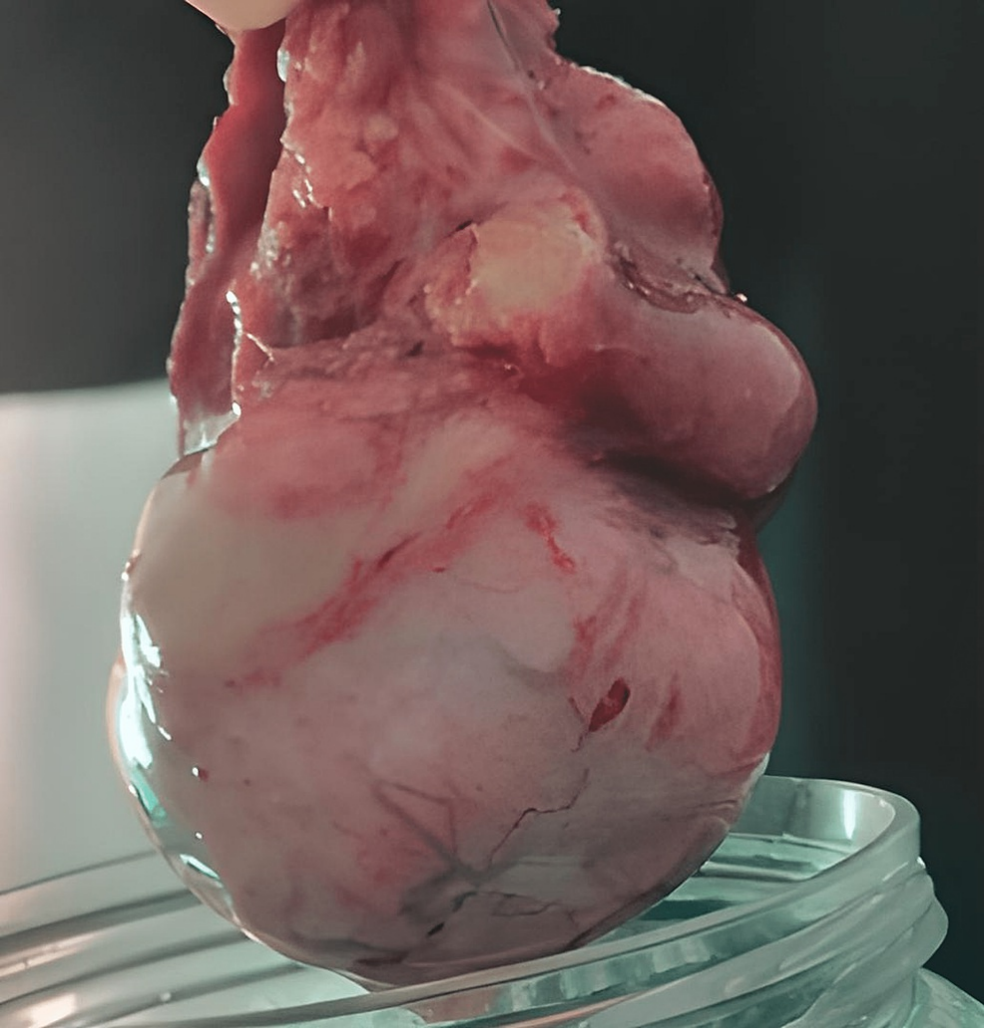
Image of the left testicle after orchiectomy

The decision was to perform a left inguinal orchiectomy with a left double J probe mount, and the patient was scheduled for surgery 48 hours later after a full biologic evaluation, including tumor markers and a thoracoabdominal-pelvic CT (Figure 3).

An anatomopathological examination revealed a partially necrotic tumor proliferation composed of lobules, tracts, and masses. The tumor cells were large, with round hyperchromatic nuclei with multiple mitoses; there was no invasion of the epididymis, the rete testis, the tunica albuginea, or the spermatic cord; the testicular cord was intact.

**Figure 4:**
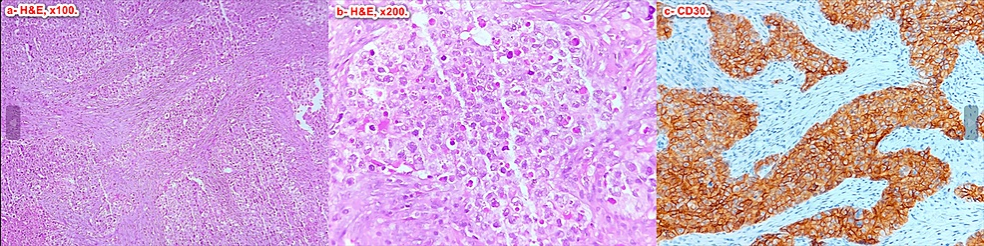
Photomicrographs of the lesion show a proliferation arranged in multiple solid nests surrounded by fibrous septa (a). Tumor cells are polygonal with atypical enlarged nuclei and abundant eosinophilic cytoplasm (b). Immunohistochemical staining shows strong positivity of tumor cells for CD30 (c).

Complementary immunohistochemistry was in favor of embryonal carcinoma (Figure 4).

The postoperative recovery period recorded no complications, and the patient was able to leave the hospital after three days. The tumor marker levels post-operatively were normal, and the patient was then sent to the laboratory for sperm preservation and to oncology for adjuvant chemotherapy.

## Discussion

Embryonal Carcinoma (EC) is a well-known type of germ cell tumor (GCT) that affects the testis and is the second most prevalent type of GCT, following Seminoma [7]. Despite its familiarity, it is surprising to note that it wasn't until 1946 that EC was officially recognized as a separate subtype of testicular neoplasm, 40 years after the discovery of Seminoma [8].

ECs primarily affects individuals in the age range of 20-30 years old [9]. Despite advancements in treatment, these tumors are more aggressive than other types of testicular germ cell tumors [9]. The high proportion of patients presenting with distant metastases is attributed to the rapid growth and early spread of the tumor through hematogenous routes [10,11]. The primary sites for hematogenous spread are the lungs, but other organs like the liver, brain, and bones may also be affected [9]. Additionally, the lymphatic invasion has been identified as a significant contributor to the spread of metastases in embryonal carcinomas, affecting mainly the retro-peritoneal para-aortic nodes and then moving on to mediastinal and supraclavicular nodes [10,11].

In our case, the Thoraco abdominopelvic CT was not delayed and was performed looking primarily for the origin of the ureter-hydronephrosis that was observed by the US, but also for possible metastatic sites in case the mass already found on the physical exam had a malignant course given the age of the patient. The obstruction that led to the ureter-hydronephrosis’s formation was due to the large left para-aortic lymph node detected by the CT-Scan. This result raised suspicion of malignancy from the testicular mass, which was confirmed later on by the anatomopathological examination and immunohistochemistry.

Given that the patient had already seen a doctor for a similar episode, a possible malignant cause was not suspected, and so the testicular mass was undiagnosed. Hence, in cases where a teenager presents with vague symptoms, it is crucial to maintain a high level of suspicion for potential tumors. Prompt diagnosis and treatment can prevent complications such as ureter-hydronephrosis.

The majority of germ cell tumors can be accurately diagnosed using light microscopy after a thorough evaluation of the morphological features that include cells with large, pleomorphic nuclei with one or more large nucleoli, dense cytoplasm that is amphophilic, poorly defined cytoplasmic membranes, numerous mitotic figures, and frequent apoptotic bodies [12]. However, in some cases, additional immunohistochemical studies may be necessary, particularly if the tumor sample is poorly fixed. The most helpful markers for identifying EC are CD30 and OCT4, although OCT4 alone does not differentiate between EC and Seminoma, unlike CD30 [7].

Treatment choices for ECs include chemotherapy and surgical resection of the mass according to whether the nature of the tumor is pure or mixed [13]. However, despite the effective initial treatment, approximately 10%-30% of patients with testicular cancer experience recurrence, typically within the first two years after achieving a complete response to treatment [14]. In a pooled analysis of 5880 patients with testicular cancer, Oldenburg et al. found late relapses in 3.2% of non-seminomas and 1.4% of seminomas [15]. Late relapses are generally observed within the first five years after treatment [14].

Late recurrences of non-seminomatous germ cell cancer are resistant to chemotherapy. The best outcome is associated with complete surgical resection in localized tumors, and this should be a crucial part of the treatment approach [15]. Given the potential difficulties in providing lifelong follow-up, it is advisable to not underestimate the possibility of late metastasis in patients with a history of embryonal carcinoma.

Our study aims to highlight the unusual clinical presentation (lumbar pain) that revealed the hidden tumor. Given that the majority of the EC masses are painless and that our patient ignored the sensation of heaviness that he reported, many other patients may relive the same story even if the mass was painful, which appeals to a raise of awareness for every clinician against these entities.

## Conclusions

Embryonal carcinoma is a well-known type of germ cell tumor that affects the testis and is the second most prevalent type of germ cell tumor, following Seminoma. It is an aggressive subtype, and it primarily affects individuals in the age range of 20-30 years old. It is often discovered by the patient or during routine medical exams as a painless or occasionally painful mass. The purpose of our study is to highlight the unusual symptom of lumbar pain that led to the discovery of the hidden tumor.

## References

[1] Kucuk S, Kucuk IG, Mizrak B (2021). Pure embryonal carcinoma of the testis in an adult male patient: case report.. Public health, economics and management in medicine.

[2] Martin A, Anderson S (2019). Combined embryonal cell carcinoma and granulomatous orchitis of the testis. J Diagn Med Sonogr.

[3] Jagtap SV, Bisht TT, Jagtap SS (2015). Pure embryonal carcinoma of testis presenting with extensive metastasis. Int J Med Sci Public Health.

[4] Bahrami A, Ro JY, Ayala AG (2007). An overview of testicular germ cell tumors. Arch Pathol Lab Med.

[5] Khan L, Verma S, Singh P, Agarwal A (2009). Testicular embryonal carcinoma presenting as chest wall subcutaneous mass. J Cytol.

[6] Iqbal S, Raiz I, Faiz I (2017). Bilateral hydroureteronephrosis with a hypertrophied, trabeculated urinary bladder. Malays J Med Sci.

[7] Kao CS, Ulbright TM, Young RH, Idrees MT (2014). Testicular embryonal carcinoma: a morphologic study of 180 cases highlighting unusual and unemphasized aspects. Am J Surg Pathol.

[8] FR NB, MO RA (1946). Tumors of the testis; a report on 922 cases. Mil Surg.

[9] Poulopoulos A, Antoniades K, Kiziridou A (2001). Testicular embryonal carcinoma metastatic to the labial mucosa of the upper lip. Oral oncology.

[10] Ayala AG, Ro JY (1998). Testicular tumors: clinically relevant histological findings. Semin Urol Oncol.

[11] Dunphy CH, Ayala AG, Swanson DA, Ro JY, Logothetis C (1988). Clinical stage I nonseminomatous and mixed germ cell tumors of the testis. A clinicopathologic study of 93 patients on a surveillance protocol after orchiectomy alone. Cancer.

[12] Bishop EF, Badve S, Morimiya A, Saxena R, Ulbright TM (2007). Apoptosis in spermatocytic and usual seminomas: a light microscopic and immunohistochemical study. Mod Pathol.

[13] Sagalowsky AI (2000). Treatment options for clinical stage 1 testis cancer. Proc (Bayl Univ Med Cent).

[14] Flora M, Costigliola A, Lavoretano S, Mollica M, Tranfa CM, Perrotta F, Calabrese C (2020). Pulmonary metastasis: very late relapse of testicular embryonal carcinoma. Monaldi Arch Chest Dis.

[15] Oldenburg J, Fossa SD (2009). Late relapse of germ cell malignancies: incidence, management, and prognosis. Methods of Cancer Diagnosis, Therapy, and Prognosis: General Overviews, Head and Neck Cancer and Thyroid Cancer.

